# Local anesthesia for transrectal ultrasound-guided biopsy of the prostate: A meta-analysis

**DOI:** 10.1038/srep40421

**Published:** 2017-01-12

**Authors:** Mingchao Li, Zhengyun Wang, Hao Li, Jun Yang, Ke Rao, Tao Wang, Shaogang Wang, Jihong Liu

**Affiliations:** 1 Department of Urology, Tongji Hospital, Tongji Medical College, Huazhong University of Science and Technology, Wuhan 430030, Hubei, China; 2 Institute of Urology, Tongji Hospital, Tongji Medical College, Huazhong University of Science and Technology, Wuhan 430030, Hubei, China; 3 Department of Respiratory and Critical Care Medicine, Tongji Hospital, Tongji Medical College, Huazhong University of Science and Technology, Wuhan 430030, China

## Abstract

A meta-analysis was performed to evaluate the efficacy of local anesthesia in alleviating pain during prostate biopsy. We searched relevant articles in PubMed and Embase. The included studies should be randomized controlled trials (RCT) using local anesthesia to alleviate pain during biopsy, which was recorded by a pain scale. Analgesic efficacy of different local anesthesia techniques were analyzed, including intrarectal local anesthesia (IRLA), periprostatic nerve block (PNB), pelvic plexus block (PPB) and intraprostatic local anesthesia (IPLA). We included 46 RCTs. PNB significantly reduced pain score compared with placebo (−1.27 [95% confidence interval [95% CI] −1.72, −0.82]) or no injection (−1.01 [95% CI −1.2, −0.82]). IRLA with prilocaine-lidocaine cream could also reduced pain (−0.45 [95% CI −0.76, −0.15]), while the IRLA with lidocaine gel was not effective (−0.1 [95% CI −0.24, 0.04]). PNB lateral to the neurovascular bundle had better analgesic effect than at prostate apex (P = 0.02). Combination use of PPB and IRLA considerably alleviated pain of patients compared with the combination of PNB and IRLA (−1.32 [95% CI −1.59, −1.06]). In conclusion, local anesthesia could alleviate patients’ pain during the prostate biopsy. PNB was not so effective as PPB.

Most prostate cancers were diagnosed by transrectal ultrasound (TRUS)-guided prostate biopsy. Although it was an efficient diagnostic method, about 65% to 90% of men felt pain or discomfort during TRUS-guided prostate biopsy^1^. In this condition, some doctors proposed that anesthetic might be a good choice to reduce pains. However, there was a dispute about anesthetic use. Previous study demonstrated that few urologists use any form of local anesthesia for TRUS biopsy^2^. Though some trials showed that use of local anesthetics made no differences^3^,^4^, many clinical studies proved apparent analgesic effect of local anesthesia compared with controls^5^,^6^,^7^. However, there has not been an exact answer about whether to use anesthetic or not up to now.

On the other hand, there were four major kinds of local anesthesia: IRLA, PNB, PPB and IPLA for prostate biopsy at the moment. Various studies have been conducted to investigate and compare the efficacy of different anesthesia methods but did not get a conclusion.

We performed this meta-analysis of RCTs about the use of anesthetic during TRUS-guided prostate biopsy to explore the analgesic efficacy of local anesthetic compared with no anesthesia or placebo, and to figure out which kind of local anesthesia was optimal.

## Results

### Search results and characteristics of included studies

**Figure 1:**
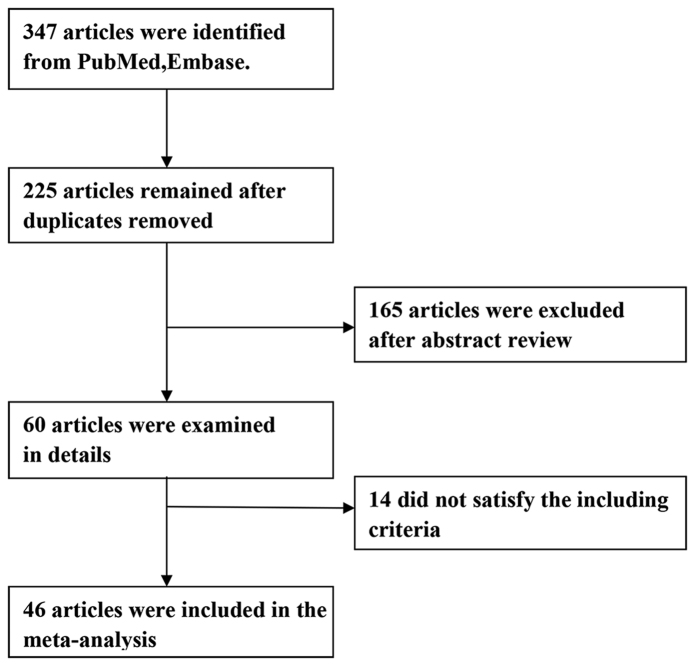
Flow diagram of trial selection process.

Our search strategy identified 347 studies in the initial database search (Fig. 1). After screening 46 RCTs^1^,^5^,^6^,^7^,^8^,^9^,^10^,^11^,^12^,^13^,^14^,^15^,^16^,^17^,^18^,^19^,^20^,^21^,^22^,^23^,^24^,^25^,^26^,^27^,^28^,^29^,^30^,^31^,^32^,^33^,^34^,^35^,^36^,^37^,^38^,^39^,^40^,^41^,^42^,^43^,^44^,^45^,^46^,^47^,^48^,^49^ met our study criteria and were included in our meta-analysis.

The characteristics of the included studies were listed in Table 1. In these studies, 42 used PNB, 19 used IRLA, 4 used IPLA, and 2 used PPB. In some studies, a local anesthesia method might be used alone or in combination with another one. PNB was used in most of the studies, but different studies chose different injection sites. We defined the different sites as base, apex and both of them. Base meant the area of neurovascular bundle at the base of the prostate, while apex was the area around the prostatic apex. Most studies used visual analogue scale (VAS) or numerical analogue scale (NAS) as the pain scale to evaluate the pain degree of patients.

### Quality of the included studies

**Figure 2:**
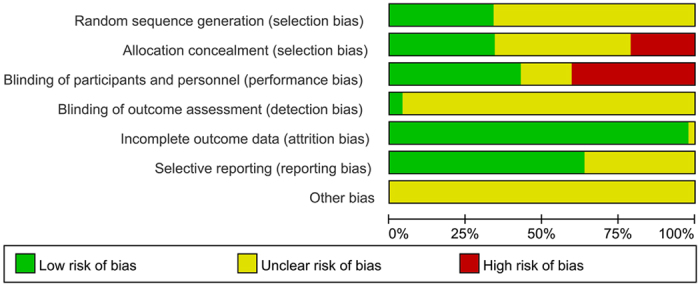
Risk of bias of included studies.

The risk of bias of included studies was presented with a risk of bias graph (Fig. 2), which showed that the quality of them was moderate. Quality of each study was shown in Supplementary Material (S1).

### Meta-analyses

**Figure 3:**
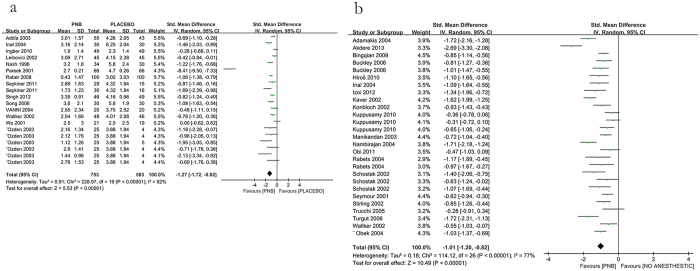
Forest plot comparing PNB with placebo and no anesthetics.

14 eligible studies showed that use of PNB significantly reduced pain compared with placebo injection (−1.27 [95% CI −1.72, −0.82], *P* < 0.00001; Fig. 3a), while 21 studies indicated that PNB could reduced pain compared with no injection (−1.01 [95% CI −1.2, −0.82], *P* < 0.00001; Fig. 3b). However, both comparisons had significant heterogeneity. We performed a sensitivity analysis by eliminating the included studies one by one. After deleting the study of Pareek *et al*.^33^ in the former comparison, *I*^2^ reduced from 92% to 59% and there were no apparent changes to the effect estimates. So this study might be the main source of heterogeneity and the reason might be that it used different pain scale. However, the sensitivity analysis could not find a study that was responsible for the heterogeneity in the later comparison. Thereby we performed a meta-regression analysis to investigate the effect of some variables (year of the study, mean age of patients, prostate biopsy numbers and dose of the anesthetics) on the heterogeneity. The result showed that the mean age of patients was apparently related to the outcomes (Table 2), so it might be a main source of heterogeneity.

**Figure 4:**
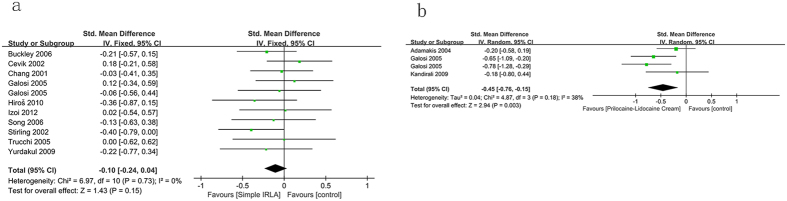
Forest plot comparing IRLA with control.

10 studies showed that use of local anesthesia with lidocaine gel made no noteworthy differences in reducing pain compared with control (−0.1 [95% CI −0.24, 0.04], P = 0.15; Fig. 4a). But 3 eligible studies indicated that use of local prilocaine-lidocaine cream significantly reduced pain compared with control (−0.45 [95% CI −0.76, −0.15], *P* = 0.003; Fig. 4b).

**Figure 5:**
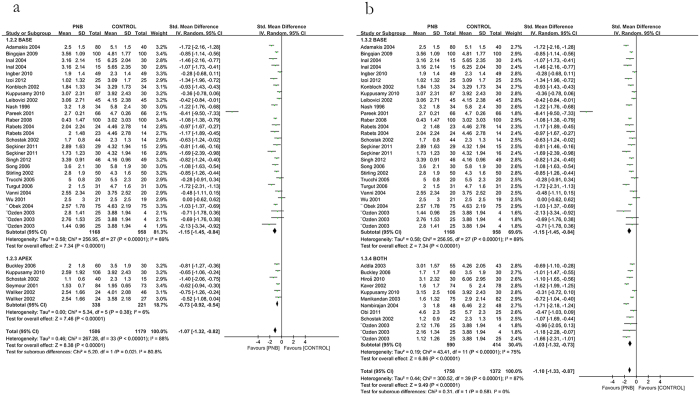
Forest plot of subgroup analysis comparing different PNBs.

The subgroup analysis of three different types of PNB showed that there were some differences between different injection sites. Compared with the PNB at prostatic apex, the PNB using the neurovascular bundles at the base of the prostate showed more effective anesthesia results (*P* = 0.02; Fig. 5a). However, no apparent difference was found between PNB with both sites and PNB at the neurovascular bundles of prostatic base (*P* = 0.58; Fig. 5b).

**Figure 6:**
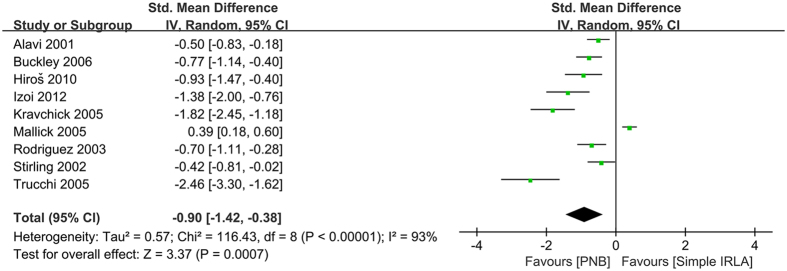
Forest plot comparing PNB with IRLA.

There were also some differences among different anesthetic techniques. The use of PNB was more efficient in reducing pain score than intrarectal anesthetic gel (−0.9 [95% CI −1.42, −0.38], *P* = 0.0007; Fig. 6).The *I*^2^ was 93% in this analysis. However, after the sensitivity analysis and meta-regression analysis we did not find any study or any parameter that might be the main source of heterogeneity.

**Figure 7:**
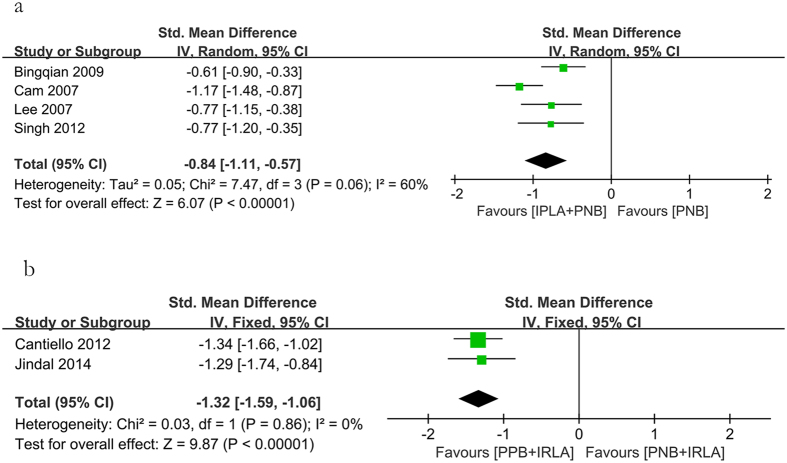
Forest plot comparing PNB with PNB + IPLA and PPB + IRLA with PNB + IRLA.

In addition, combined use of PNB and IPLA had better analgesia effect than PNB alone (−0.84 [95% CI −1.11, −0.57], *P* < 0.00001; Fig.7a). Compared with combination use of PNB and IRLA, combination use of PNB and IRLA could significantly reduce pain score (−1.32 [95% CI −1.59, −1.06], *P* < 0.00001; Fig. 7b).

## Discussion

In this meta-analysis, local anesthesia significantly alleviated pain during (TRUS)-guided prostate biopsy, except IRLA with lidocaine gel.

Although a similar meta-analysis containing 25 studies had been performed before by Tiong HY *et al*.^50^, some new studies were conducted after that and it was necessary to update it. Moreover, this meta-analysis only compared PNB with control or IRLA, while we performed more comparisons with more local anesthesia techniques and added a subgroup analysis.

PNB was the most used local anesthesia method. The first randomized, prospective study was published by Nash *et al*.^31^, showing the benefit of periprostatic local anesthesia. Our meta-analysis results suggested that PNB significantly reduced pain compared with placebo and no anesthesia, which was consistent with results from previous meta-analysis^50^.

Generally speaking, there were three different techniques of PNB: PNB lateral to the neurovascular bundle at the base of the prostate, PNB at the apex of the prostate and PNB with both regions. Our meta-analysis showed that all of the three different techniques significantly reduced pain during TRUS-guided prostate biopsy. We then performed a subgroup analysis to compare the effect of these three techniques. The results showed that anesthetic injection lateral to the neurovascular bundle was more effective than the injection at prostatic apex. But the combined injection in two sites was not superior to the single use of injection lateral to the neurovascular bundle.

The pain caused by prostate biopsy came mainly from the prostate capsule or stroma, because these areas had a rich innervation^1^. During the PNB, anesthetic infiltrated into the nerves around the prostate and blocked the nerve conduction. Hence it could decrease pain of patients. Fibers derived from the pelvic plexus traveled with vessels, forming the neurovascular bundle, and entered into the prostate at the base of the prostate just lateral to the junction between the prostate and seminal vesicle. Thereby the infiltration of local anesthesia in this region had better analgesic effect.

IRLA was a convenient local anesthesia technique and brought only a little discomfort to patients. But our results showed that IRLA with lidocaine gel could not reduce the pain during the prostate biopsy significantly. Even so, we could not deny the efficacy of IRLA. Our analysis indicated that IRLA with prilocaine-lidocaine cream could alleviate patients’ discomfort during the biopsy. This suggested that combined local anesthesia cream might have better analgesic effect than a single one. We compared the efficacy between PNB and IRLA with lidocaine gel and found that the former was more efficient in decreasing pain. It was a pity that there was not enough studies to compare PNB and IRLA with prilocaine-lidocaine cream.

Our meta-analysis also assessed two other block ways: IPLA and PPB. Mutaguchi *et al*. showed intraprostatic anesthesia was a new local anesthesia technique to anesthetize the prostate which blocked all sensory nerves from the posterior and anterior sides^51^. Due to the limited number of relevant studies, we were not able to compare the effect of PNB and IPLA alone. However, our meta-analysis suggested that the combination of IPLA and PNB had better analgesic effect than PNB alone. However, a drawback of the IPLA was that it could cause pain when penetrating the prostate capsule.

The pelvic plexus was an autonomic plexus including sympathetic and parasympathetic nerves. The midpoint of pelvic plexus located just lateral to the tip of the seminal vesicle and it was punched through by abundant branches of inferior vesicle vessels. Because the fibers innervating the prostate were derived mainly from pelvic plexus, local anesthesia in this location might be useful. In PPB, anesthesia was injected bilaterally into the pelvic plexus, therefore blocking all the nerve fibers and thus having a theoretical advantage over PNB^21^. Our meta-analysis showed that combination use of PPB and IRLA significantly reduced pain when compared with combination use of PNB and IRLA. Restricted by the number of studies, we were not able to compare the effect of PNB and PPB directly.

There were some limitations in our meta-analysis. First of all, significant heterogeneity among studies existed in some comparisons, which might reduce the reliability of our results. Even though we performed both sensitivity analysis and meta-regression analysis to investigate the source of heterogeneity, not all the heterogeneity source could be found. Hence we used the random effect model in our analysis. In addition, there was not enough number of studies in some comparisons. More studies were expected to reinforce our results.

In summary, it was the first meta-analysis about the role of PPB and intraprostatic anesthesia in reducing pain during TRUS-guided prostate biopsy to our knowledge. Our meta-analysis suggested that local anesthesia such as PNB, PPB, IPLA and local prilocaine-lidocaine cream was effective in alleviating pain for TRUS-guided prostate biopsy. Besides, PNB lateral to the neurovascular bundle at the base of prostate had better analgesic efficacy than PNB at the prostatic apex. It was also revealed that PPB might be more effective than PNB. Hence, PPB was potential to be a standard of care for patients undergoing TRUS-guided prostate biopsy.

## Methods

### Study search

We searched Pubmed and Embase for all papers, including conference abstracts, in any language published before May 1, 2016. Our search strategy was: (prostate biopsy) and ((local anesthesia) or analgesic) and (pain or discomfort) and (randomized or randomization). Reviews and nonhuman studies were not included. In addition, if two studies were conducted by the same authors and parts of their patients were also the same, only the latest one with more patients was included. The search was conducted by two authors separately.

### Inclusion criterion

The studies that met the following criteria were included: (1) RCTs; (2) patients underwent TRUS-guided prostate biopsy with local anesthetic; (3) local anesthetic was compared with placebo or no anesthetic group, or different kinds of local anesthesia methods were compared; (4) pain during the biopsy should be recorded by a pain scale.

### Data Extraction

All available RCTs that had data about pain during TRUS-guided prostate biopsy were selected for analysis. The major characteristics of included articles were extracted: the first author, the year of publication, study design, the number of patients and groups, the utilized local anesthesia methods and their location and the pain scale.

The mean and standard deviations of pain scores were extracted to perform the analysis. These data were recorded by different pain scales, such as VAS, NAS and others. We extracted the pain scores which were taken immediately at the end of the biopsy for evaluation. If a research used both placebo injection and no analgesic, both groups were used as controls and the patients’ number in the anesthesia group was divided equally into two parts. Similarly, if more than one local anesthesia groups was used in one study, the number of patients of the control group was divided equally by the number of anesthesia groups. All the data were extracted independently by different study authors and any discrepancy was resolved by consensus.

### Quality assessment

Quality assessment of the included studies was performed by The Cochrane Collaboration’s tool for assessing risk of bias, including assessments of random sequence generation, allocation concealment, blinding of participants and personnel, blinding of outcome assessment, incomplete outcome data, selective reporting and other bias.

### Statistical analysis

All meta-analyses were performed using RevMan 5.2 (The Nordic Cochrane Centre, the Cochrane Collaboration, 2012, Copenhagen, Denmark). Continuous outcomes were presented as standardized mean difference (SMD) with 95% confidence interval (CI). Statistical heterogeneity was assessed with the *I*^2^ statistic, in which*I*^2^ > 50% was considered to be of high heterogeneity. When significant heterogeneity was present, data were analyzed using the random effect model and a sensitivity analysis or meta-regression analysis was performed to find the source of heterogeneity. The meta-regression analysis was performed by using Stata 12.0. Differences were considered statistically significant when *P *< 0.05.

Firstly, we compared the outcomes of PNB groups with placebo groups and no anesthesia groups separately. Secondly, we analyzed the anesthesia efficacy of different IRLA methods, including the simple IRLA with lidocaine gel and IRLA with lidocaine-prilocaine cream. And then, we performed subgroup analysis to compare the efficacy of PNB methods with different injection positions. At last we compared the outcomes of different kinds of local anesthesia methods.

## Additional Information

**How to cite this article**: Li, M. *et al*. Local anesthesia for transrectal ultrasound-guided biopsy of the prostate: A meta-analysis. *Sci. Rep.* **7**, 40421; doi: 10.1038/srep40421 (2017).

**Publisher's note:** Springer Nature remains neutral with regard to jurisdictional claims in published maps and institutional affiliations.

## Supplementary Material

Supplementary Files

## Figures and Tables

**Table 1:** Characteristics of included studies.

First Author	Year	Study Design	Patients Number/Groups	Local Anesthesia Methods	Injection Site of PNB	Pain Scale
Alavi AS	2001	RCT	150/2	PNB;IRLA	Base	VAS
Adamakis I	2004	RCT	198/3	N;IRLA;PNB	Base	VAS
Addla SK	2003	RCT	98/2	P;PNB	Base + Apex	VAS
Akdere H	2013	RCT	80/2	N;PNB	Between Base and Apex	VAS
Bingqian L	2009	RCT	300/3	N;PNB;PNB + IPLA	Base	VAS
Buckley MR	2006	RCT	240/4	N;IRLA;PNB	Base, Apex	NAS
Cam K	2008	RCT	200/2	PNB;PNB + IPLA	Base	VAS
Cantiello F	2012	RCT	180/2	IRLA + PPB;IRLA + PNB	Base	VAS
Cevik I	2002	RCT	100/2	P;IRLA		VSA
Chang SS	2001	RCT	108/2	P;IRLA		VAS
Galosi AB	2005	RCT	210/4	N;P;IRLA		VAS
Hiros M	2010	RCT	90/3	N;IRLA;PNB	Base + Apex	VAS
Inal G	2004	RCT	90/3	N;P;PNB	Base	VAS
Ingber MS	2010	RCT	50/2	P;PNB	Base	VAS
Izol V	2012	RCT	100/4	N;PNB;IRLA	Base	VAS
Jindal T	2014	RCT	139/3	IRLA;IRLA + PPB;IRLA + PNB	Base	VAS
Kandirali E	2009	RCT	80/4	N; Perianal; IRLA; Perianal + IRLA		VAS
Kaver I	2002	RCT	152/2	N;PNB	Base + Apex	VAS
Knobloch R	2002	RCT	68/2	N;PNB	Base	VAS
Kravchick S	2005	RCT	114/4	N; PNB; Perianal	Base	VAS
Kuppusamy S	2010	RCT	386/4	N;PNB	Base	VAS
Lee HY	2007	RCT	152/3	IPLA + P;PNB + P;IPLA + PNB	Base	VAS
Leibovici D	2002	RCT	90/2	P;PNB	Base	VAS
Mallick S	2005	RCT	356/2	IRLA;PNB	Base	VAS
Manikandan R	2003	RCT	235/3	N;PNB	Base + Apex	VAS
Nambirajan T	2004	RCT	96/2	N;PNB	Base + Apex	VAS
Nash PA	1996	RCT	64/2	P;PNB	Base	Other
Obek C	2004	RCT	300/4	N; PNB,IRLA + PNB	Base	NAS
Obi AO	2011	RCT	75/3	C;PNB	Base + Apex	VAS
Ozden E	2003	RCT	175/7	P;PNB	Base, Base + Apex	VAS
Pareek G	2001	RCT	132/2	P;PNB	Base	Other
Raber M	2008	RCT	300/3	P;PNB;IRLA + PNB	Base	VAS
Rabets JC	2004	RCT	75/3	N;PNB	Base	VAS
Rodriguez A	2003	RCT	96/2	IRLA;PNB	Apex	VAS
Schostak M	2002	RCT	170/4	N;PNB	Base, Apex	VAS
Seçkiner I	2011	RCT	90/3	P;PNB	Base	VAS
Seymour H	2001	RCT	157/2	N;PNB	Apex	Other
Singh SK	2012	RCT	142/3	P;PNB;PNB + IPLA	Base	VAS
Song SH	2006	RCT	90/3	P;IRLA;PNB	Base	VAS
Stirling BN	2002	RCT	150/3	N;IRLA;PNB	Base	Other
Trucchi A	2005	RCT	60/3	N;PNB	Base	Other
Turgut AT	2006	RCT	93/3	N; PNB	Base	VAS
Vanni AP	2004	RCT	40/2	P;PNB	Base	VAS
Walker AE	2002	RCT	121/3	N;P;PNB	Apex	Other
Wu CL	2001	RCT	40/2	P;PNB	Base	VAS
Yurdakul T	2009	RCT	100/4	N;IRLA;IRLA + PNB	Base	VAS

RCT: randomized controlled trial; N: no anesthetic; P: placebo; PNB: periprostatic nerve block; IRLA: intrarectal local anesthesia; IPLA: intraprostatic local anesthesia; PPB: pelvic plexus block; Base: neurovascular bundle at the base of the prostate; Apex: the apex of prostate; VAS: visual analogue scale; NAS: numerical analogue scale.

**Table 2:** Meta-regression of moderators in the comparison between PNB and no anesthesia group.

Moderator	Study Number	β	95%CI		P	R2
Year	27	−0.008	−0.069	0.052	0.78	−0.056
Age	24	0.214	0.092	0.335	0.001*	0.421
Anesthetic dose	27	0.002	−0.067	0.072	0.942	−0.054
Biopsy number	27	−0.081	−0.167	0.006	0.066	0.095

PNB: periprostatic nerve block.

*significant when P < 0.05.
