# Supplementary material for: Local anesthesia for transrectal ultrasound-guided biopsy of the prostate: A meta-analysis
Source: Sci Rep. 2017 Jan 12;7:40421. doi: 10.1038/srep40421 (PMC5227686; doi:10.1038/srep40421)
Supplement: Supplementary Files [file srep40421-s1.pdf]

Local anesthesia for transrectal ultrasound-guided biopsy of the prostate: A meta-analysis

Mingchao Li, Zhengyun Wang, Hao Li*, Jun Yang, Ke Rao, Tao Wang, Shaogang Wang, Jihong Liu

	Random sequence generation (selection bias)	Allocation concealment (selection bias)	Binding of participants and personnel (performance bias)	Blinding of outcome assessment (detection bias)	Incomplete outcome data (attrition bias)	Selective reporting (reporting bias)	Other bias
¨ Obek 2004	?	−	−	?	+	+	?
¨Ozden 2003	?	−	−	?	+	?	?
Adamakis 2004	?	?	?	?	+	+	?
Addla 2003	+	+	+	+	+	?	?
Akdere 2013	?	?	−	?	+	+	?
Alavi 2001	+	+	−	?	+	+	?
Bingqian 2009	+	+	?	?	+	+	?
Buckley 2006	?	?	+	?	+	?	?
Cam 2007	?	?	+	?	+	+	?
Cantiello 2012	+	+	+	?	+	?	?
Cevik 2002	?	?	+	?	?	?	?
Chang 2001	?	?	+	?	+	+	?
Galosi 2005	?	?	?	?	+	?	?
Hiroš 2010	?	?	−	?	+	+	?
Inal 2004	?	?	−	?	+	?	?
Ingber 2010	?	?	+	?	+	?	?
Izoi 2012	?	?	−	?	+	?	?
Jindal 2014	+	+	+	?	+	?	?
Kandirali 2009	?	?	−	?	+	?	?
Kaver 2002	+	+	+	?	+	?	?
Konbloch 2002	?	?	−	?	+	+	?
Kravchick 2005	+	+	−	?	+	?	?
Kuppusamy 2010	?	?	?	?	+	?	?
Lee 2007	+	+	+	?	+	?	?
Leibovici 2002	+	+	+	?	+	+	?
Mallick 2005	?	?	−	?	+	+	?
Manikandan 2003	?	−	−	?	+	+	?
Nambirajan 2004	?	−	−	?	+	?	?
Nash 1996	?	?	+	?	+	+	?
O¨bek 2002	?	−	−	?	+	+	?
Obi 2011	+	+	?	+	+	?	?
Pareek 2001	?	?	+	?	+	+	?
Raber 2008	+	+	+	?	+	+	?
Rabets 2004	?	−	−	?	+	+	?
Rodriguez 2003	?	?	?	?	+	+	?
Schostak 2002	+	+	?	?	+	+	?
Seçkiner 2011	+	+	+	?	+	+	?
Seymour 2001	?	−	−	?	+	+	?
Singh 2012	+	+	+	?	+	+	?
Song 2006	?	−	−	?	+	+	?
Stirling 2002	+	+	?	?	+	+	?
Trucchi 2005	?	−	−	?	+	+	?
Turgut 2006	?	?	+	?	+	+	?
Vanni 2004	?	?	+	?	+	+	?
Wallker 2002	?	?	+	?	+	+	?
Wu 2001	+	+	+	?	+	+	?
Yurdakul 2009	?	−	−	?	+	+	?
